# Supplementary material for: CD133 Expression in Circulating Tumor Cells as a Prognostic Marker in Colorectal Cancer
Source: Int J Mol Sci. 2025 May 15;26(10):4740. doi: 10.3390/ijms26104740 (PMC12111574; doi:10.3390/ijms26104740)
Supplement: Supplementary file 1 [file ijms-26-04740-s001.zip › ijms-3572863-supplementary.pdf]

Figure S1a Stage I（44cases）

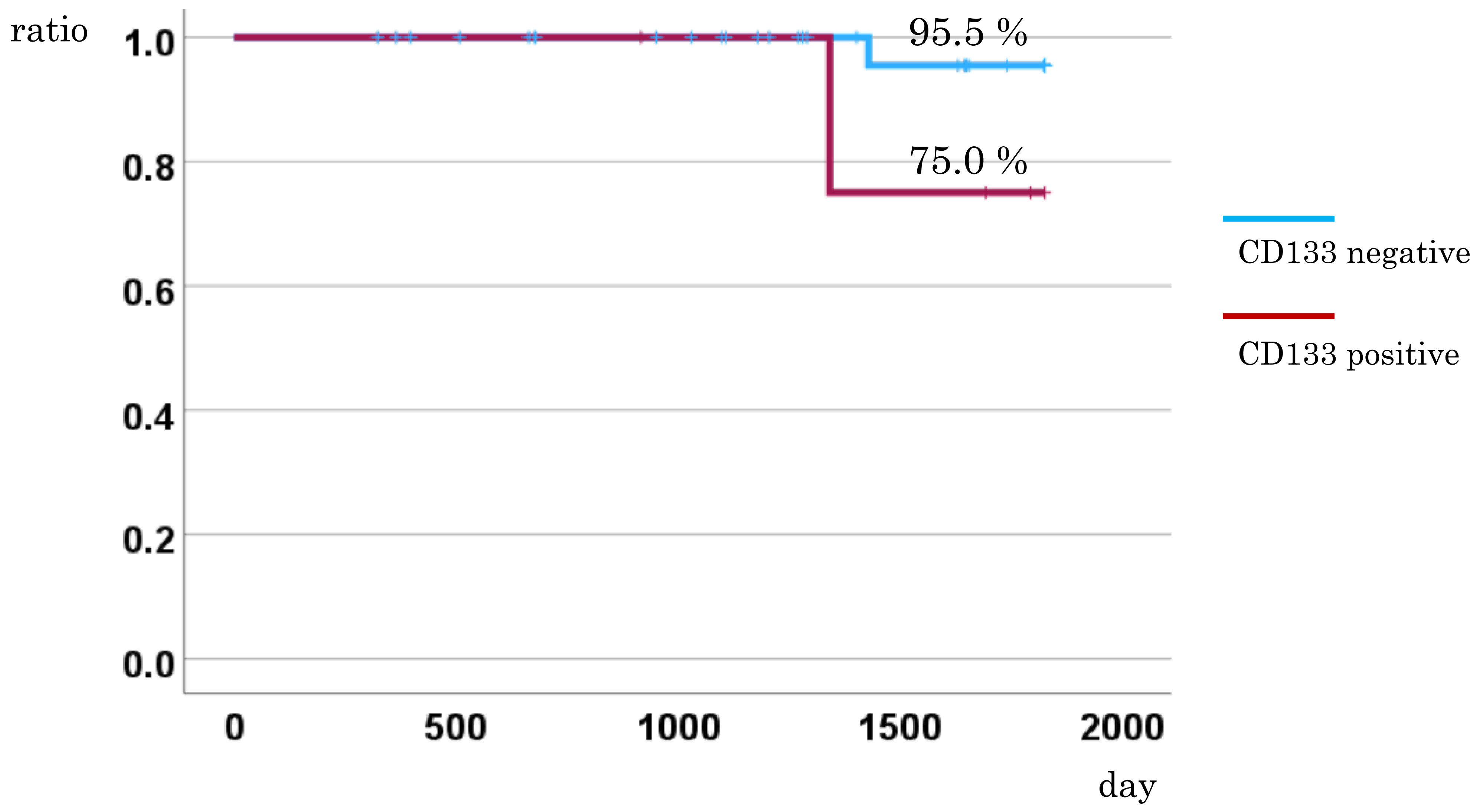

Figure S1b Stage II（64cases）

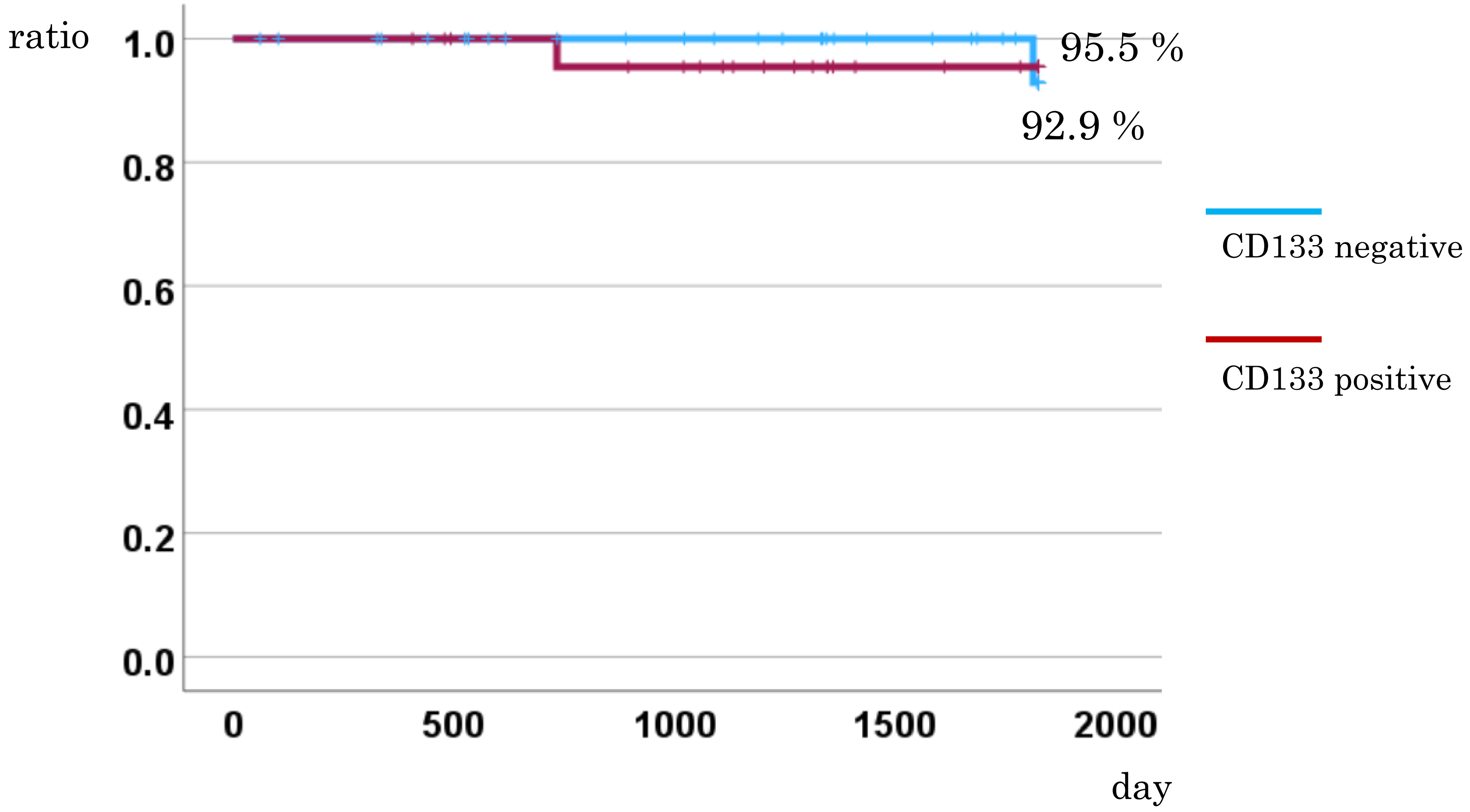

Figure S1c Stage IV（33 cases）

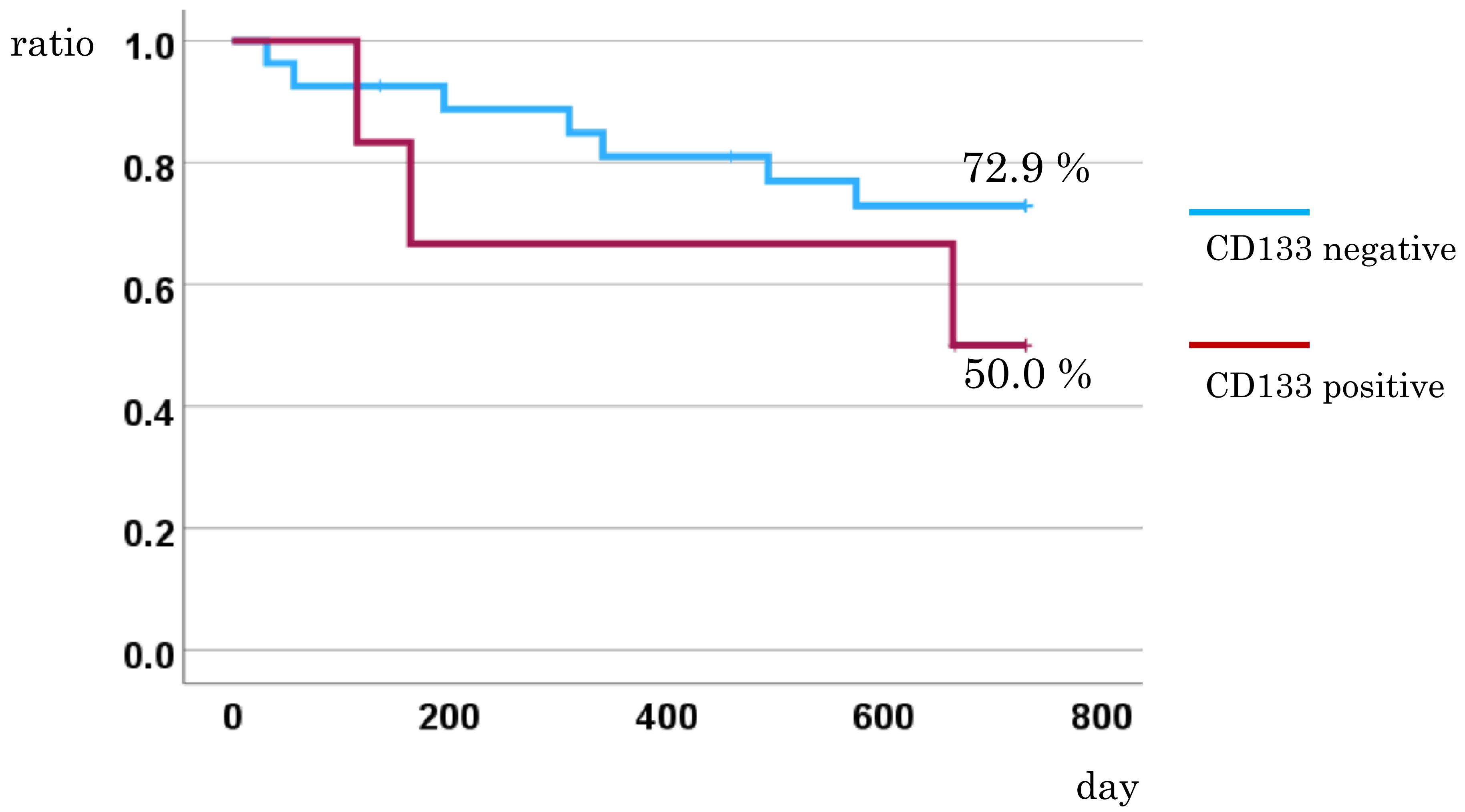

Figure S1d Stage III（54cases）

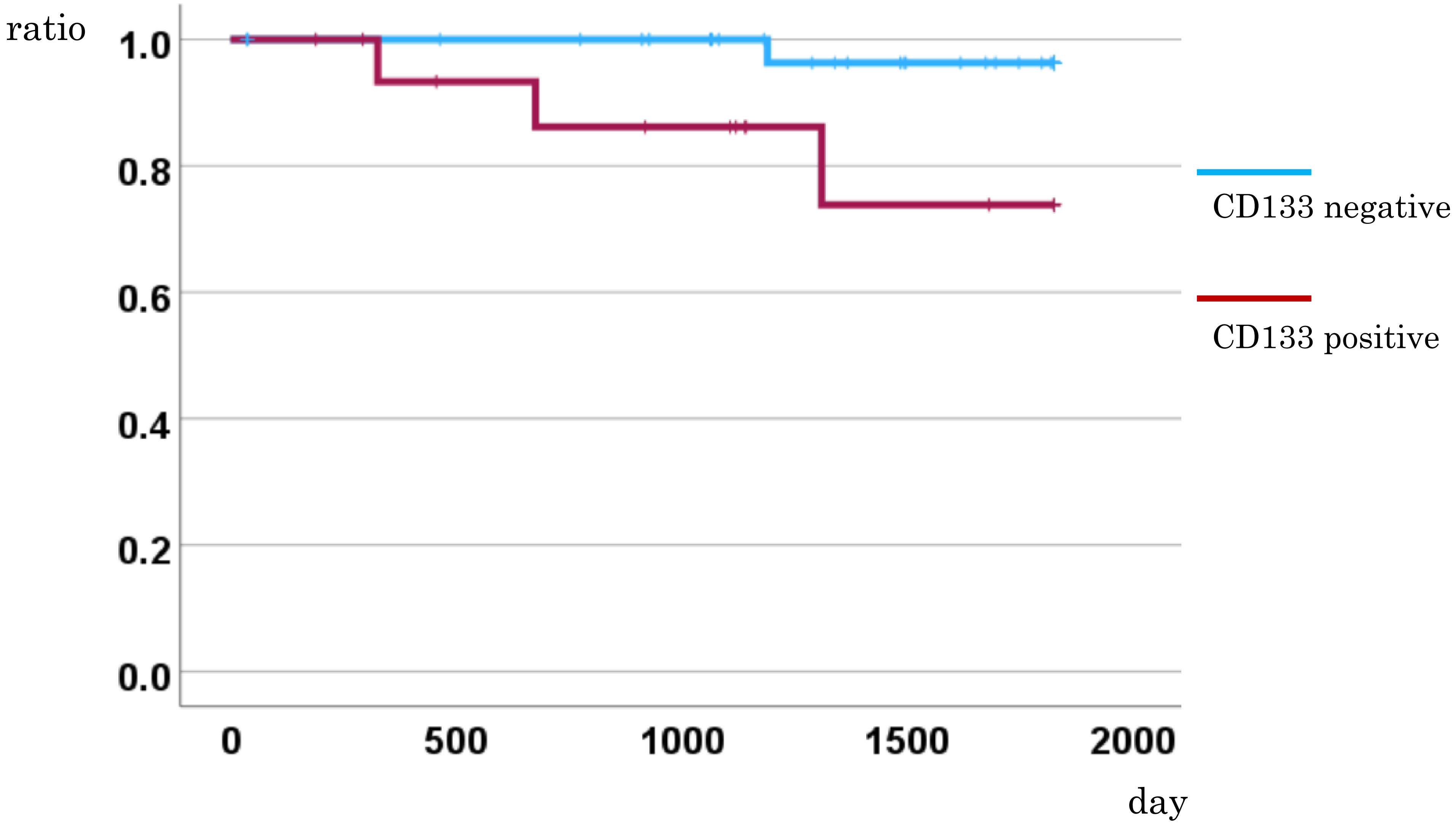

Figure S2a

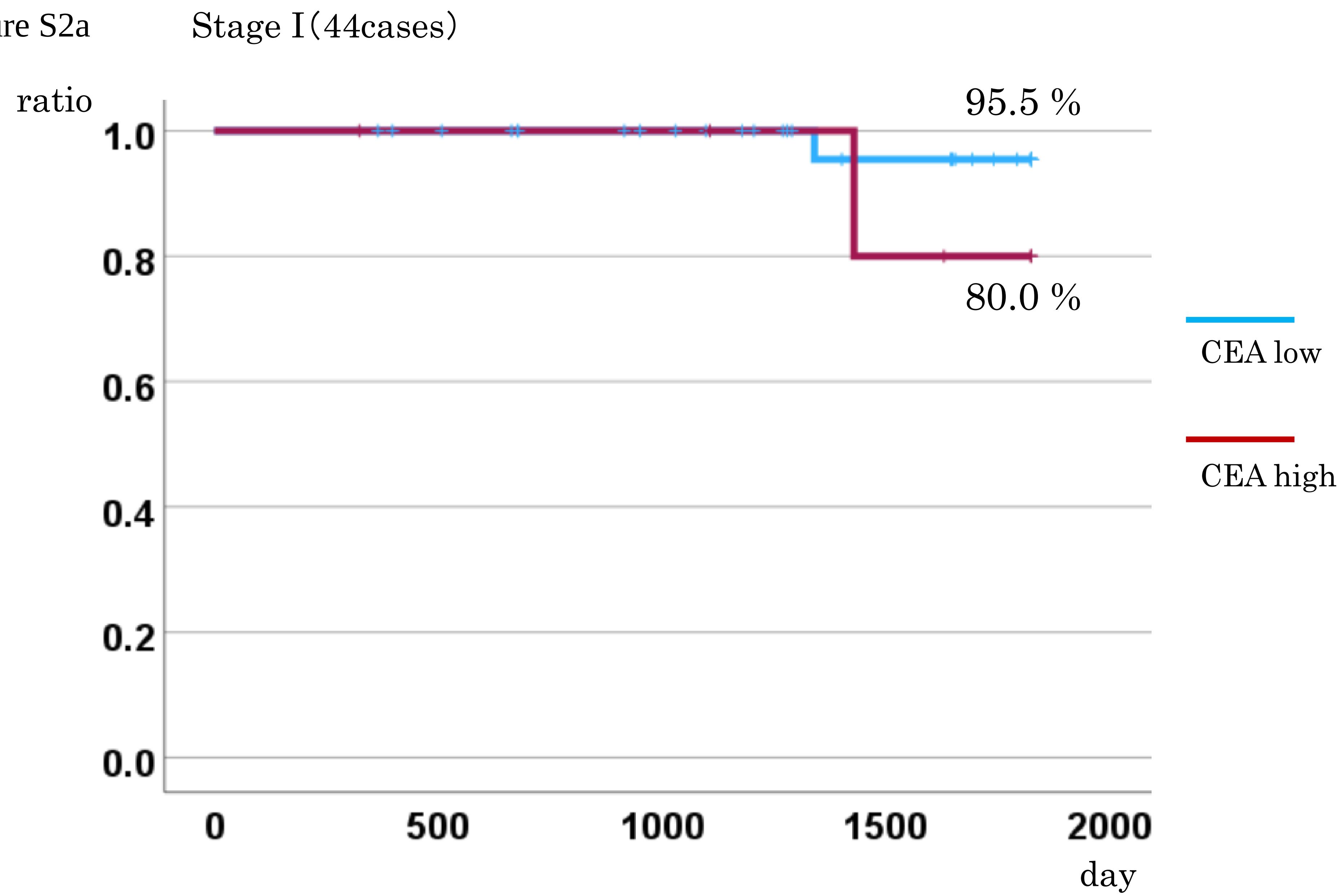
Stage I(44cases)
ratio
1.0
0.8
0.6
0.4
0.2
0.0
0
500
1000
1500
2000
day
95.5 %
80.0 %
CEA low
CEA high

Figure S2b Stage IV（33cases）

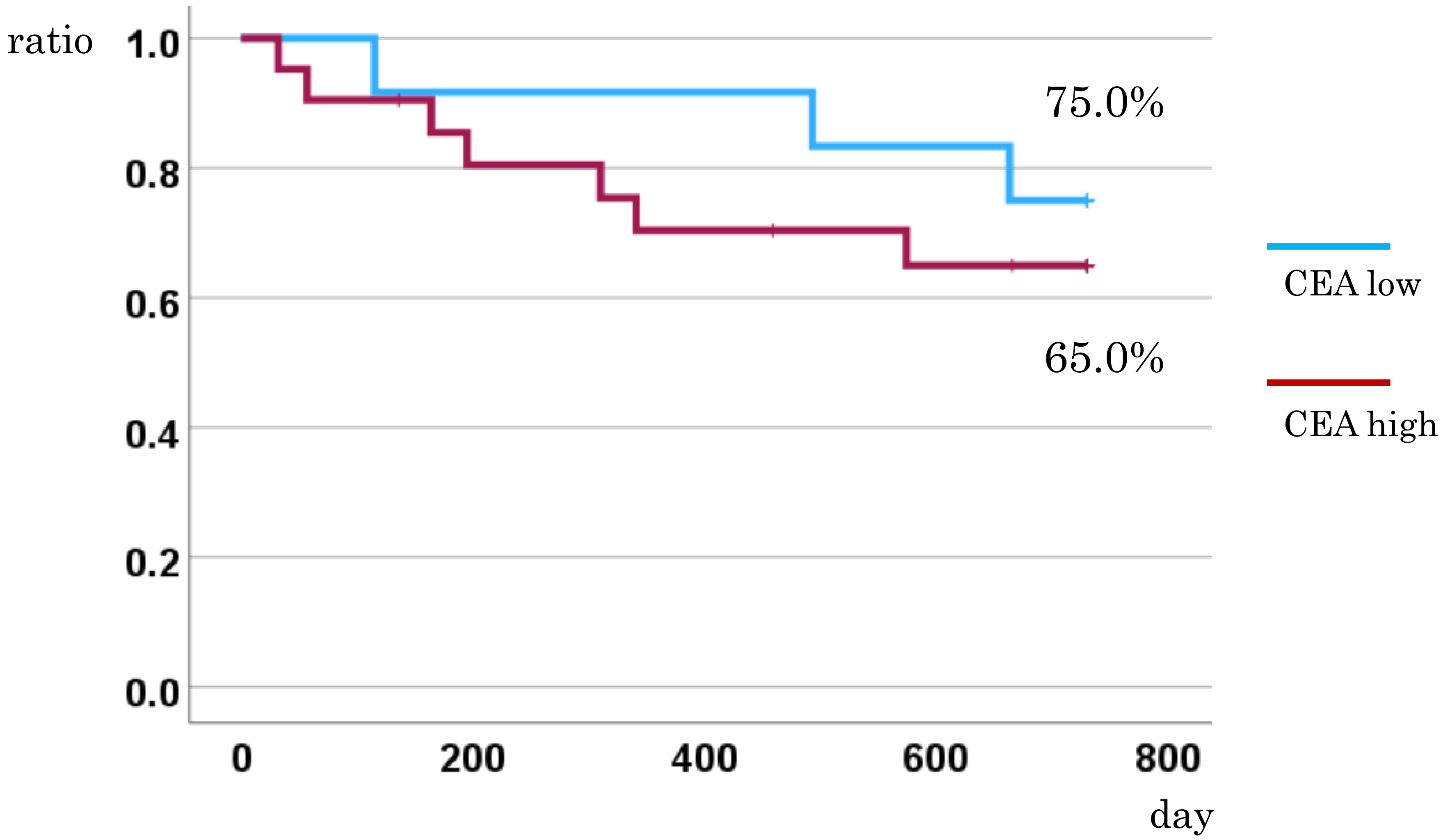

Figure S2c Stage II（64cases）

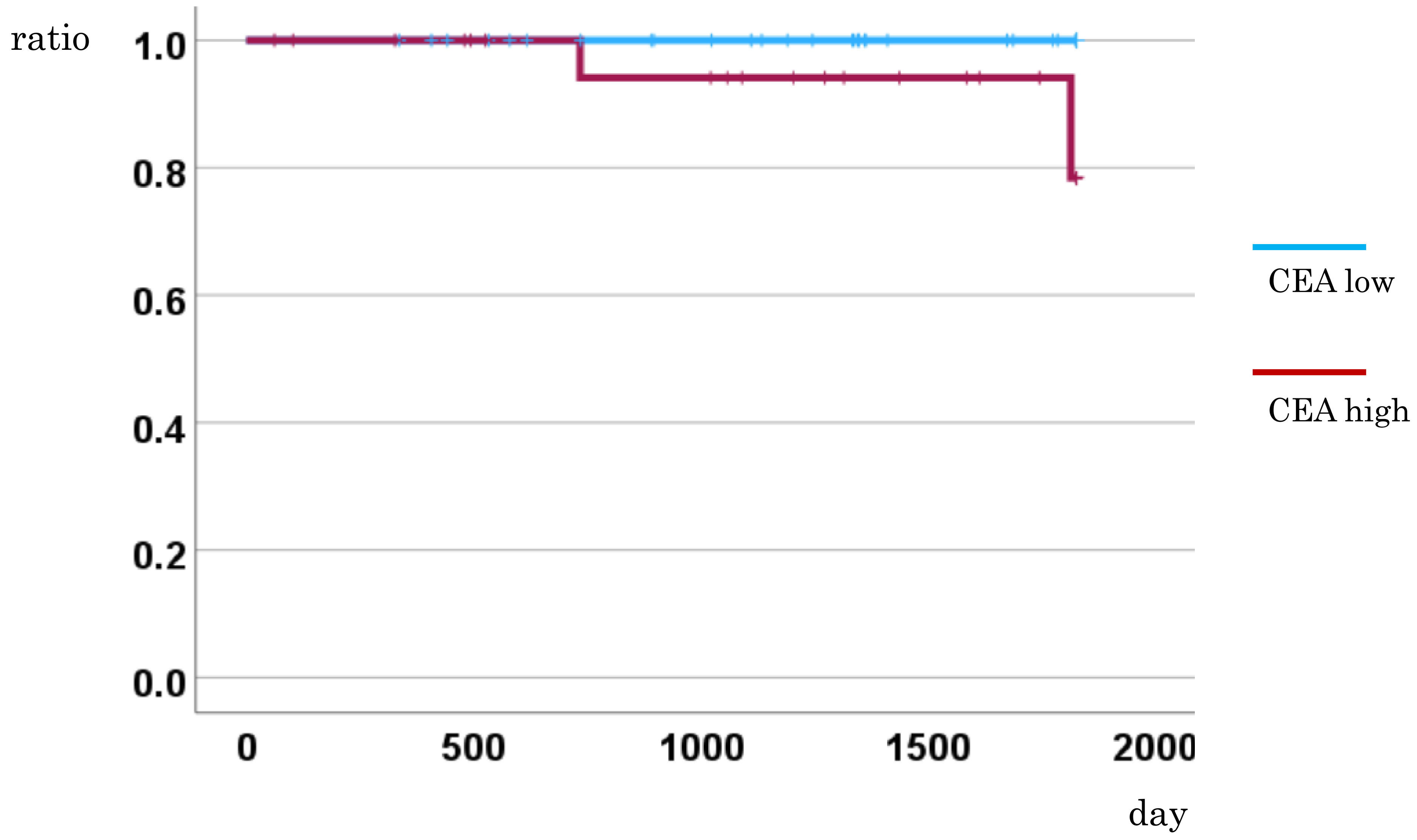

Figure S2d Stage III (54cases)

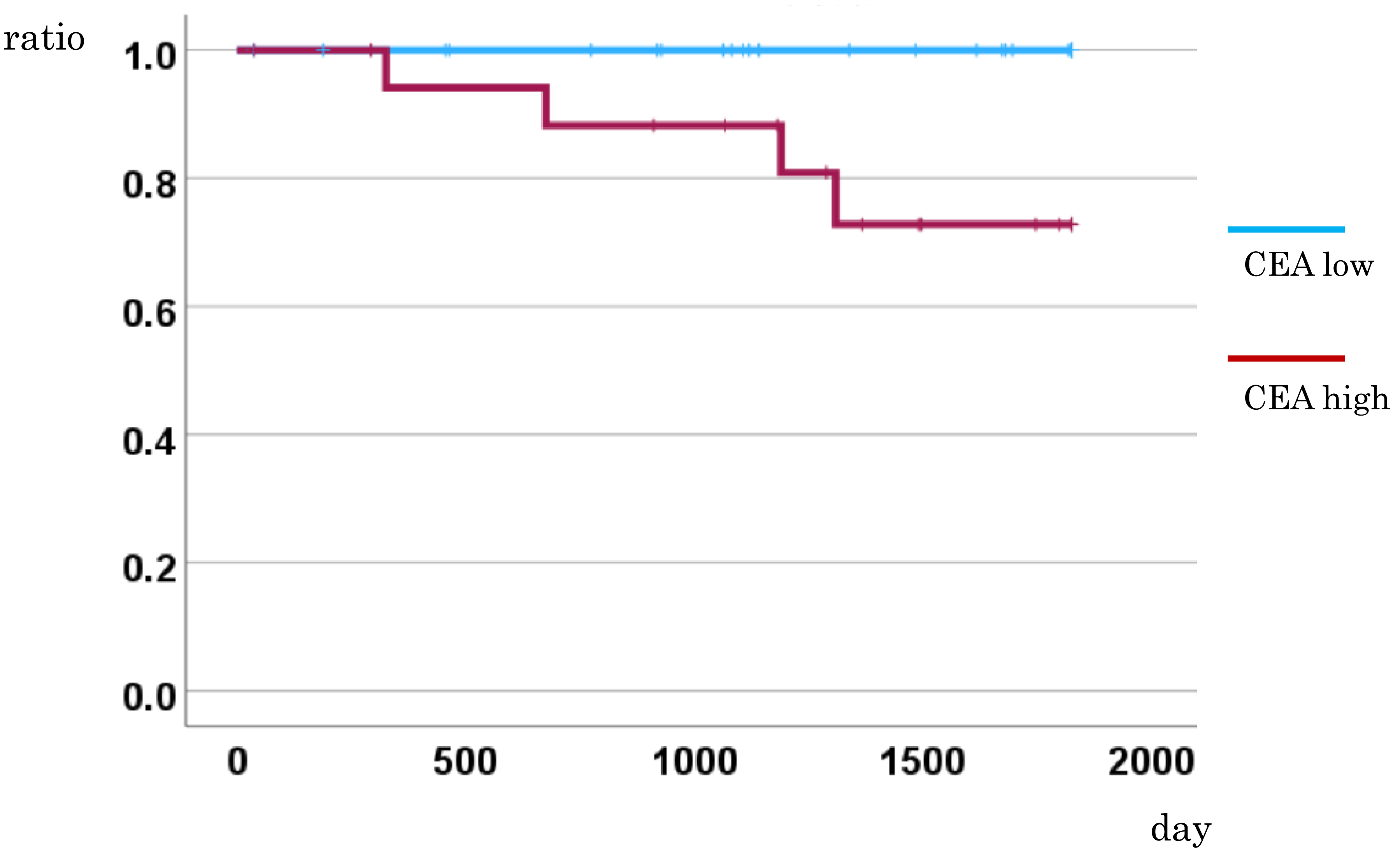

Figure S3a Stage II（64cases）

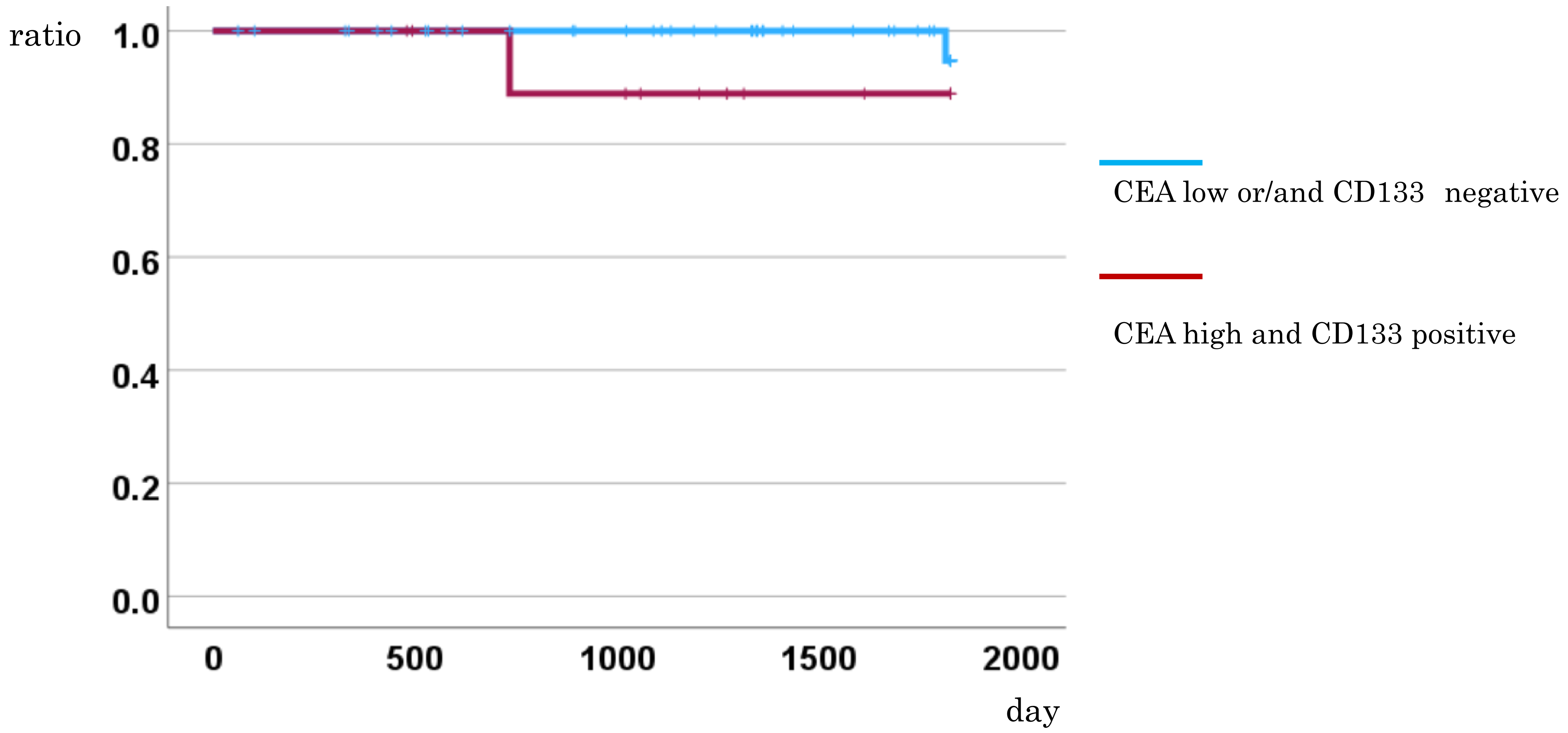

Figure S3b Stage III（54cases）

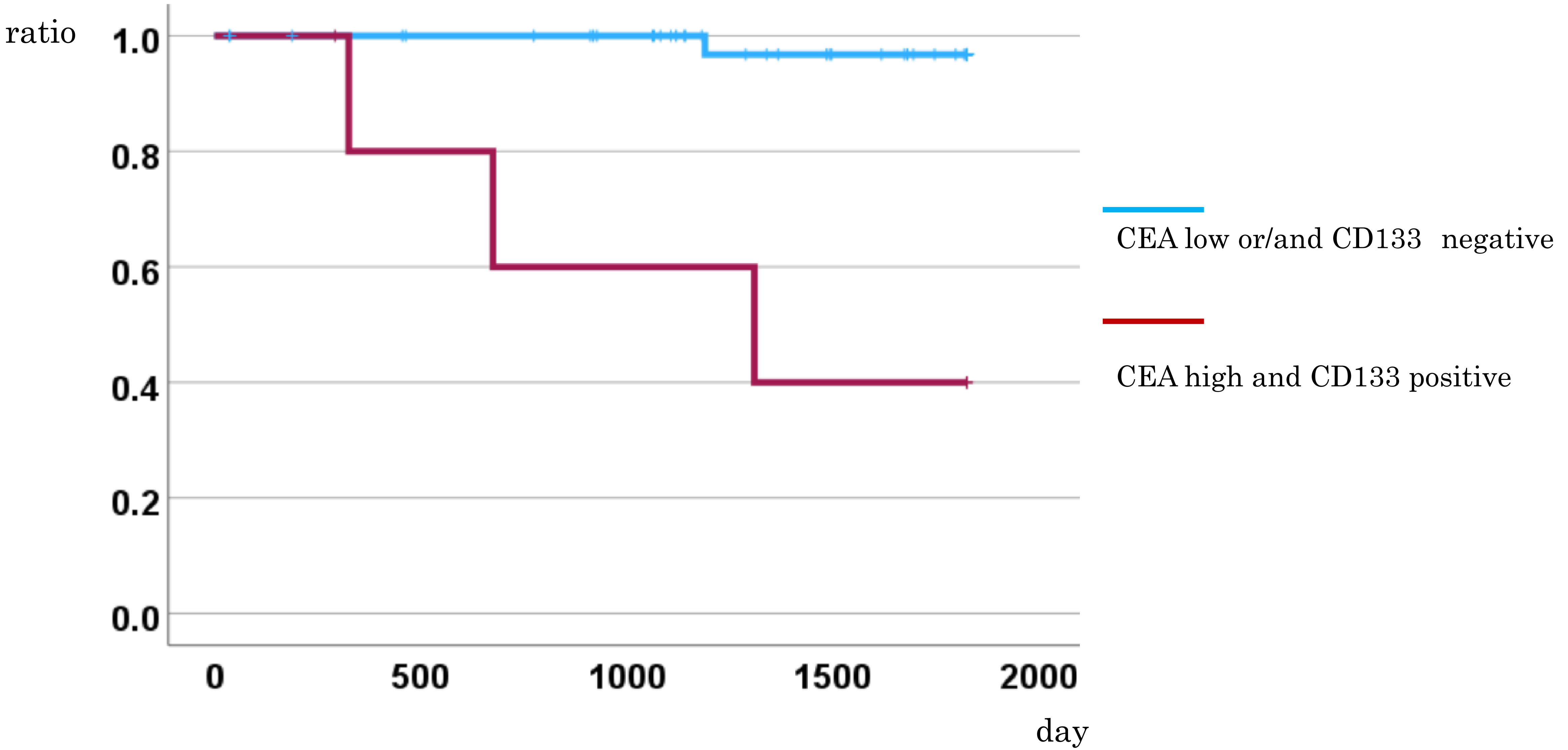

Captions of all SM Figures

Figure S1. (a) 5-year DSS for CD133-positive and CD133-negative cases in stage I, (b) 5-year DSS for CD133-positive and CD133-negative cases in stage II, (c) 2-year DSS for CD133-positive and CD133-negative cases in stage IV, (d) 5-year DSS for CD133-positive and CD133-negative cases in stage III.

Figure S2. (a) 5-year DSS for cases with low and high CEA levels in stage I, (b) 2-year DSS for cases with low and high CEA levels in stage IV, (c) 5-year DSS for cases with low and high CEA levels in stage II, (d) 5-year DSS for cases with low and high CEA levels in stage III.

Figure S3. (a) Comparison of the 5-year DSS between cases with CD133-positive CTCs and high CEA levels and other cases in stage II, (b) comparison of the 5-year DSS between cases with CD133-positive CTCs and high CEA levels and other cases in stage III.
